# NATIVITY DIFFERENCES IN OLDER MEXICAN AMERICAN HOSPITALIZATIONS: STRENGTH AND FUNCTION AS MARKERS

**DOI:** 10.1093/geroni/igae098.3457

**Published:** 2024-12-31

**Authors:** Juan Ventura, Soham Al Snih

**Affiliations:** The University of Texas Medical Branch, Galveston, Texas, United States; University of Texas Medical Branch in Galveston, Galveston, Texas, United States

## Abstract

Hospitalizations are a difficult occurrence for many older adults. Additionally, health differences have been noted in adults according to their country of origin. This study aims to compare nativity differences in hospitalizations using handgrip strength (HGS) and short physical performance battery (SPPB) among Mexican American older adults. This prospective cohort study included 1,324 Mexican Americans aged ≥75 years from the Hispanic Established Population for the Epidemiological Study of the Elderly. Measures included socio-demographics, body mass index, multi-morbidity (≥2 medical conditions), depressive symptoms, disability, cognitive function, and HGS–SPPB performance groups. Generalized estimating equation models were utilized to estimate the odds ratio (OR) and 95% Confidence Interval (CI) of hospitalization as a function of HGS and SPPB performance groups. Foreign-born participants in the high HGS–low SPPB (OR=0.62, 95% CI=0.40-0.97) and high HGS–high SPPB (OR=0.42, 95% CI=0.18-0.97) groups experienced significantly lower odds of hospitalizations compared to those in the lowest performing group (low HGS–low SPPB). No association was found between HGS-low SPPB (OR=0.71, 95% CI=0.50-1.00) and HGS-high SPPB (OR=0.63, 95% CI=0.36-1.11) with hospitalizations among US-born. Those with Multimorbidity and falls had greater odds of hospitalizations among both US and foreign-born. High levels of education were associated with lower odds of hospitalization among foreign-born and high depressive symptoms were associated with greater odds of hospitalizations among US-born. Overall, older Mexican Americans who performed high in HGS and high in both HHS and SPPB experienced relatively decreased odds of hospitalizations, with the effect being significant in those who were foreign-born.

